# Fever—Contagion—Quarantine

**Published:** 1825-01-01

**Authors:** 


### MEDICO-CHIRURGICAL

### REVIEW.

VOL. VI.] Analytical Series. [No. 19.

" Nec tibi quid liceat sed quid fecisse decebit
" Occurrat, mentemque domat respectus honesti."

VOL. II.] JANUARY 1, 1825. [No. 3.

[NEW SERIES.]

### I.

### FEVER—CONTAGION—QUARANTINE.

1. *Official Report on the Fever which appeared on Board H. M. Ship Bann, on the Coast of Africa, and amongst the Detachment of Royal Marines, forming the Garrison, on the Island of Ascension, in the Year* 1823. By WILLIAM BURNETT, M.D. one of the Commissioners of the Medical Department of His Majesty's Navy—Physician in Ordinary to His Royal Highness the Duke of Clarence,—and Honorary Fellow of the Imperial Medico-chirurgical Academy of St. Petersburgh. 8vo. pp. 78. London, July 1824.

2. *A comparative View of Fever and Inflammatory Complaints; with Essays, illustrative of the Seat and Nature of Fever.* By THOMAS MILLS, M.D. Licentiate of the King and Queen's College of Physicians. 8vo. pp. 125. Dublin and London, June 1824.

3. *Evils of Quarantine Laws, and Non-Existence of Pestilential Contagion; deduced from the Phenomena of the Plague of the Levant—the Yellow Fever of Spain—and the Cholera Morbus of India.* By CHARLES MACLEAN, M.D. 8vo. pp. 444. London, 1824.

4. *Observations on Fever.* By R. WADE, Member of the Royal College of Surgeons, and Apothecary to the Westminster General Dispensary. 8vo. pp. 83. London, 1824.

5. *Medical Report of the Fever Hospital and House of Recovery, Cork Street, Dublin, for the Year* 1823. By WM.

STOKER, M. D. Senior Physician of that Hospital, &c. 8vo. pp. 101. Dublin and London, 1824.

THE multiplied and ever multiplying objects which are presented, in quick and endless succession, to the mind's eye of the public, render it impossible that the impression left by any one of them can have more than a momentary duration in the memory. It is astonishing indeed—and somewhat mortifying to human vanity,—to observe how soon events, which shake empires, and absorb, at the time, every feeling of almost every individual in the civilized world, sink into oblivion, and, in a few months, are only remembered when some chronicle is opened that records the historical narration. The battle of Waterloo is now scarcely brought to recollection, even by the sight of the hero who won it—and the Duke of Wellington rides through the streets of the metropolis without a single finger being pointed at the conqueror of Buonaparte! Who is it that calls up to his memory the once dreaded and adored name of Napoleon? The traveller who admires his magnificent and stupendous road over the Semplon—or traces in amazement the perilous route of his army over the snow-clad summit of the Great St. Bernard!—If this be the fate then, of such great and eventful transactions, how can the minor events of literature or science expect to retain possession of the public mind, for more than a moment? In our own profession, if any one subject could be quoted as an exception to this general rule, it would be FEVER. Although its very existence has long been denied in this and in some other countries, yet it has continued, down to the present time, to intrude itself most pertinaciously on the attention of the profession, and to excite controversies, discussions, and literary warfare that appear interminable.—But even FEVER, the most universal of all diseases, has not been able to excite uninterrupted interest in the minds of the faculty. It is only at those periods when its destructive ravages are felt or apprehended, that the subject occupies afresh the public curiosity, and sets the press at work to evolve the jarring opinions of practitioners respecting the nature, causes, and treatment of each successive epidemic. The West India islands, the United States of America, the swamps of Walcheren, the vallies and ports of Spain, the fortresses of Malta and Gibraltar, the distant shores of Batavia, the jungly banks of the Ganges, the mountains of Ceylon—and lastly, the United Kingdom of England, Ireland, and Scotland, have, in our own days, furnished ample materials for etiological, pathological, and therapeutical controversies, that have not yet ceased—and are still very far from having settled the question on any one of the above important points of discussion. But, notwithstanding the discrepancies of opinion and practice that still prevail respecting fever, there is, we believe, a nearer approach to unanimity of doctrine and uniformity of treatment, at the present than at any former period since the dogmas of the schools gave way to clinical observation, pathological research, and unshackled discussion. The discrepancies too, are less than they seem. It is principally the contending parties who come before the public—the great majority of that public are pretty nearly agreed on the very points which give rise to so much controversy.

The works at the head of this article bear upon some interesting points of the etiology, pathology, and treatment of fever; but they will not amalgamate, and we must take them into consideration separately.

Dr. Burnett's official report relates to the severe visitation of fever, from which the crew of the Bann and the small garrison of Ascension suffered such an extraordinary loss of lives in the year 1823. It bears also on the question of *contingent contagion*, on which so much has been written, pro and con, proving, we think, in a satisfactory manner, that fevers, even within the tropics, are capable of taking on, under particular circumstances, a character of contagion which had nothing to do with their original production. We shall endeavour to lay before the public a succinct, but we hope, clear account of this fatal visitation.

The Bann, sloop of war, stationed on the coast of Africa, anchored off Free Town, Sierra Leone, on 11th January 1823, continuing there till the 25th March, her crew in perfect health. On the 25th March, one case of fever, with severe inflammatory symptoms, was put on the list, and terminated fatally on the 30th of the same month.

*March 26th.* The master and two seamen were attacked with fever, and recovered.

*27th.* The Bann sailed from the coast.

We must now go back a little to state that, on the 19th of March, the Bann sent her pinnace on board a merchant timber vessel, called the *Caroline*, to assist her in coming to an anchor. This vessel had sailed from Malta on the 26th September 1822, and arrived at Sierra Leone on the 3rd November, in perfect health, as appears by the official report of Mr. Shower, colonial surgeon, and Dr. Barry of Sierra Leone. She proceeded up the river on the 12th and 13th November—loaded her cargo of wood at Tomboo island, 30 miles above Free Town—lying the whole of the time in a low swampy situation, surrounded with mangroves. About a month after she came here, she became sickly, as might be expected, and sent eight men, with the usual remittent fever of the country, to the hospital. Of these, four died. On the 28th January she drove from her anchors and stranded, but was soon got off. On the 11th February she got down to the anchorage off Free Town, having grounded on rocks and shoals, in moving down, and become leaky. She anchored about three miles from the squadron, and endeavoured to procure invalids to navigate her home. On the 22d February (nearly three months after her arrival on the coast) the Caroline sent a man to the hospital with the concentrated form of the fever. *At this time*, Dr. Barry of the hospital observes in his official report, the fever "had begun its ravages on shore." He consequently and expressly rejects the idea of the fever being communicated to the colony by the Caroline. On the 13th March, the said Caroline anchored near the mouth of the river, in Cockle bay; but the leak increasing, she returned to the anchorage off Free Town on the 19th March, with her crew much weakened by incessant labour at the pumps and constant exposure to the sun. Some of her men were again taken ill, and some ran away from her. It was at this time that the Bann, as was before-mentioned, gave her assistance. About the 22d or 23d of March also, a survey was held on the Caroline by the master of the Bann, the master of the Snapper, and the carpenter and carpenter's mate of the Owen Glendower frigate. They remained on board the Caroline several hours, visiting the hold and other parts of the ship. Of all these officers, and of the boat's crews accompanying them, only one man was taken ill of the fever—namely, the master of the Bann, who suffered an attack, but was only two or three days on the sick list, from the 26th to the 28th March. It may be observed also, that the crew of the Caroline had constant communication with the boats' crews of the different men of war, at the watering place, without any accident occurring. Finally, this same Caroline sailed for England on the 5th May, where she arrived on the 29th June, without losing a man on the voyage, though several invalids and others took passage in her from the coast.

It is also proper to state, that, in September 1822, the Bann captured a Spanish schooner (the San Raphael) laden with slaves of Whydabb, which schooner arrived in the river of Sierra Leone in the following December. On board of this vessel was a party of men from the Bann, but they suffered nothing while conducting the slaves to the colony. Afterwards, however, the said schooner was dispatched with a lieutenant and 40 men to reconnoitre the Gallinas. On this cruise, four of her men were seized with fever, but none died. She returned, and anchored near the Caroline—before the fever appeared in the Bann. According to the testimony of the surgeon of the Bann, the fever, by which they were so severely scourged, appeared first in the schooner San Raphael, where her small detachment of men suffered more in proportion than the crew of the Bann. Out of the first eight or nine who were seized in the Bann, four had been employed in the schooner—and three (two marines and a carpenter) had never been out of the Bann on any duty. The schooner was cleared of Europeans, and taken with the Bann on her voyage to Ascension.

The above digression from the narrative of the Bann was necessary, in order that the reader might be enabled to appreciate the value of that hypothesis (for really it seems little more) which places not only the fever in the Bann, but that which ravaged the colony of Sierra Leone to the account of the Caroline timber vessel!

What dire mishaps from trivial causes spring!

We now return to the Bann. She sailed from Sierra Leone on the 27th March 1823. Between that date and the 31st three more men were seized with fever. On the 3d April, four were added to the list, and from this time the fever spread rapidly till almost the whole of the ship's crew were attacked. Of ninety-nine who underwent the fever, thirty-four died—or full one in three!—She reached Ascension on the 25th April, in a dreadful state, as may be supposed—fifteen of the thirty-four having already died. Tents were immediately pitched, at the distance of nearly five hundred yards from the garrison, the sick (45 in number) landed—and all intercourse interdicted.

The island of Ascension is well known to be a volcanic rock, less than twenty miles in circumference, with some green patches of verdure on its more elevated parts. The destination of Napoleon to the neighbouring island of St. Helena, changed the aspect of Ascension from a place of occasional turtle turning to a small garrison of thirty-five officers and men. These occupied three posts, namely, the barrack at the beach—the Springs, about four miles from the barracks—and the Green Mountain, 2500 feet above the level of the sea.

"It has been said," says Dr. Burnett, "that this island was always particularly healthy, previous to the appearance of the fever, which followed the Bann's visit to it in 1823. This statement is evidently erroneous, for I find, on reference to the Journals of the medical officers, who at different times had charge of the garrison before the appearance of the late epidemic, an abstract from which is in the Appendix, that not only has dysentery and hepatitis been very prevalent, as well as occasional attacks of fever, but likewise that a fever, called the bilious remittent, in the year 1818, attacked almost every man on the island, which the assistant surgeon (see Appendix, No. IV.) attributes to an unusually wet turtle season, when the men are much exposed, by watching at night, to turn those animals. Moreover, there is in the Journal of Mr. Robert Malcolm, for 1818, a case of this disease, which commenced on the 1st of June, and terminated by death the next day, with all the symptoms of yellow suffusion and black vomit, &c. (see Appendix, No. V.) which are said peculiarly to characterize the yellow fever; and having shown this case to the surgeon of the Bann, now in London, he declares it to be exactly similar to the cases of fever which lately proved so fatal in the Bann, and amongst the marines at Ascension. It also appears, from the abstract above mentioned, that out of one hundred and thirty-two cases of disease, which are recorded in these Journals, twelve died and nineteen were invalided; and, though perhaps all the fatal cases are inserted in the Journals, it is well known that those documents seldom contain above a third of the cases which actually occur." p. 11.

At the time of the Bann's arrival, however, the garrison was in good health. About two days after the landing of the Bann's sick, one case of fever occurred, but soon recovered. The surgeon of the garrison dates the commencement of the fever at eighteen days from the landing of the sick seamen, viz. 11th May, when a boy was violently attacked and ultimately died. Up to this time, it appears that the restrictions between the garrison and sick sailors were little more than nominal. Several articles were landed from the Bann into the garrison, and some of the officers of the island had gone on board the Bann to bid at the sale of the effects of the deceased!—These were restrictions with a vengeance! Their eyes were now opened, when it was too late; and every precaution was taken to prevent the disease being communicated to the outposts. In spite of these, fifteen individuals were taken ill at the Springs—but fortunately none at the Green Mountain, though one of the men belonging to that post had been on board the Bann. After the Bann's fever had nearly ceased, it went on spreading in the garrison, and of twenty-eight men taken ill fifteen died—or more than one half! Besides these, two boys and four women belonging to the marines of the island paid the forfeit of their existence.

While the Bann was at Ascension, the Driver sloop of war arrived from Sierra Leone, where she had been under the same circumstances as the Bann, yet with her crew in perfect health. Soon after her arrival, two clerks were sent from her on board the Bann—they were both taken ill—and one of them died.—Two other officers who *visited* the Bann, escaped. As two or three cases of fever (one of them fatal) shewed themselves about this time on board the Driver, the captain put off to sea, and no more fever appeared. There are yet other proofs of contagion to come forward. The Bann sailed from Ascension on the 2d of June, and arrived at Bahia, on the coast of Brazil, on the 10th of the same month. In the meantime a number of relapses and eighteen new cases of fever occurred on board of her.—Here she found the Creole, Tartar, Briton, and Doris frigates. A boat from the Tartar came alongside, during a heavy rain, and the boat's crew sheltered themselves on board. In a few days a number of these were seized with the fever and some of them died. In fact, it appears that the fever extended to forty-five of the Tartar's crew, of whom five died. The disease, however, appears to have been of a milder character, and no mention is made of yellow suffusion. The other ships of the squadron at Bahia continued in perfect health. Again; a boy entered on board the Bann at Bahia, from a merchant vessel, three days after which, he had a most violent attack of the fever. At this place the Bann's hold was examined, and found, in every respect clean—"*but no examination was made under the limber boards, where all the mischief was found to be, on examining the hold of the Pyramus, which ship had suffered about a year before, so severely in the West Indies, from yellow fever.*" p. 17. Before making any remarks of an etiological nature, we shall introduce the following extract from Dr. Burnett's report, bearing on the symptoms and *post mortem* appearances.

"It will be sufficient to observe, that the disease commenced with symptoms of high excitement, and a yellow appearance of the eyes, followed in many cases by vomiting of bilious matter, and by irritability of stomach; frequently by diarrhœa, after the first cathartic, and by great depression of strength, dark yellow suffusion, and death. Captain Phillips mentions, in his letter to me, that two fatal cases had coffee-coloured vomiting; and I have reason to think that this occurred in more instances after he left the island; but many had neither that symptom, nor indeed any vomiting or irritability of stomach whatever.

"The morbid appearances found on *post mortem* examinations, were chiefly confined to the mucous coat of the stomach and intestines, though many of the neighbouring parts showed marks of inflammation; the former generally contained a large quantity of a liquid of a dark colour different from bile. The villous coat in many parts slightly inflamed, and the whole lined with a white glairy mucus. The same appearances extended more or less to the intestines, and the vessels of the mesentery were enlarged. The treatment consisted, in the beginning, of liberal evacuation by the lancet, and purgatives, and in the progress of the disease, calling in the aid of various other remedies, such as calomel, the cold affusion, blisters, &c. often without any benefit. I must, however, observe, that as far as I can judge, no remedies were better calculated to meet this truly formidable and intractable disease. I am far, however, from advocating the indiscriminate or unmeasured use of the lancet, though I have no hesitation in saying, that, when employed early and judiciously, it is one of the most certain and powerful means of arresting or controlling fever that we are acquainted with; and it is only justice to add, that in my opinion, the medical officers performed their duties not only with ability,* but with the utmost attention and humanity.—Nothing would be more unfair than to regard them, from the mortality that took place, as having failed in the former: let any unprejudiced person look at the character of the disease, displayed in its symptoms, rapid progress, and morbid appearances, after death, and point out a better plan of treatment, not from the closet in England, but at the bed-side of the patients, under such circumstances, as those gentlemen had to contend with."† p. 13.

We now come to two interesting questions—was this fever in the Bann contagious? Where did it originate? We think that few men not completely blinded by prejudice, or wedded to some favourite doctrine, will reject or doubt the evidence which has been brought forward, respecting the contagious character which the fever evinced in the Bann, both at Ascension and Bahia. If this evidence be questioned, it is in vain to look for farther testimony in human affairs. But the other question, of the origin of the fever, is by no means so easily disposed of. Four sources of the fever may be conjectured or suspected—1st. The climate of the coast, and the ordinary causes of disease—2d. Contagion from the Caroline—3d. Ditto from the San Raphael—4th. Local causes in the ship's hold.

1. The *first* cause is that which the surgeon of the Bann accuses, in conjunction with intemperance on the part of the crew—and much exposure to the sun while refitting the ship and her tender the San Raphael. It may be objected to this source, that the other ships at the same anchorage continued healthy. This, at first sight, appears insuperable. But if we recollect that a very slight difference in the *discipline and occupation* of a crew, will make all the difference between sickness and health, the objection loses much of its force, and the surgeon's opinion is far from unreasonable.

* The author of the "Select Dissertations" justly observes, at page 285, that "this disorder, like all others of a pestilential and malignant nature, sets at defiance the art of physic in its curative capacity; human skill, of which the most anxious and judicious exertions have not been wanting, has been found of little avail in abating the fatality of this epidemic."

† Dr. Barry says, in his Medical Report from Sierra Leone, for 1823, "the only patients who escaped were those in whom *early* venesection was had recourse to."

2. That either the Bann or the Colony received the fever from the Caroline, after the circumstances which we have detailed, will not be credited by any man in his senses (Sir Gilbert Blane excepted); and therefore we shall not waste words on this topic.

3. The San Raphael Tender appears, on a superficial glance, to be a much more probable source of infection than the Caroline;—and one of our respected cotemporaries* seems to have come to the conclusion that the febrific miasm was generated by the slaves (themselves escaping) and propagated afterwards among the Europeans both in the Tender and the Bann. But let us recollect that the European navigating crew of the San Raphael prize were on board, *along with the slaves,* from September till sometime in December, and returned to the Bann *in perfect health*—that afterwards, another English crew were put on board of her, while she was sent up the river Sierra Leone thirty miles to have her bottom repaired, which crew suffered no sickness—that still longer afterwards, she was sent out on a cruize off the Galinas with a lieutenant and forty men, where she had only four men sick, none of whom died, and all of whom, with the crew, returned well or convalescent to the Bann—and finally, that it was after all this, that her little crew became sickly while lying at anchor in the same place with the Bann—will it be believed the febrific miasm of the slaves now at last came into action and gave rise to the tragic scene that ensued? We cannot bring ourselves to such a conclusion.

4. It has now been well ascertained, from fatal experience, that while we are seeking for external sources of contagion, we have the enemy in our own bosom. Between the timbers and planks in the holds of vessels miasmata have been generated that scattered destruction among the unsuspecting crews above. The Pyramus was a recent and melancholy example. To this source we are strongly inclined to attribute the fever in the Bann, aided by the atmospheric exposure and intemperance alluded to by the surgeon. It is not a little in favour of this local origin that the fever began forward in the ship and came gradually to the after part, till nearly all the officers and men were affected. Still, however, it must be confessed, that much obscurity hangs over the origin of the fever, both in the Colony and in the Bann, which we need not now expect to remove.

Dr. Burnett professes to belong to the moderate, and we believe more numerous party, who believe that yellow fever, so called, is generally originated from other causes than contagion,

* Vide London Medical Repository, No. 8, p. 145. August 1824.

though it may and does assume a contagious character occasionally, under peculiar circumstances. In elucidation of these positions Dr. Burnett brings forward several authentic documents, which have never before been made public. Of these we shall take notice of two or three.

1. The Bedford, of 74 guns, sailed from St. Fiorenzo Bay, in the island of Corsica, on the 29th July, with several other men of war, and arrived at Gibraltar on the 24th August, her crew in perfect health. Here they caulked the ship, *and the shingle ballast was shifted in the hold.* On Sunday, the 6th September, when the ship's company was mustered, there was not a man on the sick list;—but in the course of one week, from this period, they sent upwards of 130 to the hospital with yellow fever.

"The general symptoms," says the Surgeon in his Journal, "were a cold and hot paroxysm, quickness of pulse, head-ache, nausea, general and great debility, and, in two-thirds of the cases, local pains in some parts of the chest. The debility, in many cases, was so great, that sentinels at their post, and sailors in the common duty of the ship, fell down as if struck with lightning, without any previous complaint whatever, and as far as I was able to remark, the danger was in proportion to the degree of this symptom. On the second or third day of the disease, all their skins acquired more or less of a yellow tinge; and in the worst cases (at the hospital) were of as deep a dye as any thing I have ever seen—the yellow fever of the West Indies not excepted. Eleven died at the hospital before we left Gibraltar (24th of September), and others were left dangerously ill." p. 30.

Here three things may be observed:—*First,* The fever was accompanied by yellow suffusion, as deep as in the worst cases of the West India yellow fever. *Secondly,* There was no assignable cause for it, excepting the shifting of the ballast in the hold. *Thirdly,* It evinced no contagious character on board or on shore—because accumulation was not permitted, and cleanliness and ventilation were enjoined. This fever in the Bedford is quoted by Sir Gilbert Blane (Select Dissertations, p. 288). Though the real object of the quotation does not appear very clear.

2. His Majesty's ship Kent (74) had been cruizing off Toulon, from the 29th April till the 26th June, when she anchored in Port Mahon harbour, crew healthy. From thence she sailed on the 1st July, with a convoy for Gibraltar. On the 6th, 7th, 8th, and 9th of July, 170 men were taken ill with fever, and the sick list increased on the passage to, and at Gibraltar.—There were in all 240 cases, of whom eleven died. Yellow suffusion was universal, and the surgeon of the ship denominates the fever—"*the causus icterodes or ardent yellow fever.*"

The sick being distributed into empty transports prevented an accumulation which would doubtless have proved very deleterious. Even as it was, the surgeon states that the fever evinced a contagious character in some instances on board the transports, after the sick had been removed into them. The cause of this fever, in all probability, might be fairly traced to febrific agents in the harbour of Port Mahon.

We shall now take a short glance at another quarter of the globe.

3. The Pyramus frigate sailed from Plymouth, and arrived at Antigua, where she lay thirty-four days in English Harbour. After this she went to several other places, returning to Barbadoes on the 9th November. The fever on board of this ship, is said first to have broken out in English Harbour, Antigua, and daily increased—a considerable number having died in the hospitals of Antigua and Barbadoes, where the sick were landed. A sea cruize had no effect in mitigating the disease. On the contrary, the fever went on increasing, and therefore on the 14th January the provisions and stores were removed, by slaves, at Antigua. "Yet six weeks after every person had been landed, it was a common occurrence for six or seven to be taken ill daily, arising, as it may be presumed, from their frequent communication with the sick." At length, the limber boards in the hold were taken up, when such an offensive smell issued forth as caused fainting in some on the spot, and several of the officers attending on this duty were immediately seized with the fever.

"The state of the hold, under the limber boards, is compared by Staff surgeon Hartle, to a bog of the most pestiferous nature on shore; and Mr. Comrie mentions, that three or four of the slaves employed in cleaning the ship were attacked with the fever. I may here observe, that previous to the sailing of the Pyramus from England, she had had her magazine fitted on a new plan, and that the shavings and chips resulting therefrom, instead of being removed from the ship, were unfortunately suffered to remain in the lower part of the hold, and thus mixed with the bilge water under the limber boards.

"Dr. Fitzgerald states, that some time previous to the appearance of the fever, the foul state of the hold was sufficiently indicated by smells of a very disagreeable nature issuing therefrom, and diffusing themselves over the ship, and that a candle, when placed at 'the mouth of the hold,' was immediately extinguished; and I was also informed by an officer of the ship whom I formerly knew, and whom I lately saw in the royal hospital at Haslar, that all the captains of the hold had lost their lives." p. 38.

Dr. Burnett states his conviction that the fever on board the Pyramus had nothing to do with the injection of coal-tar, as was at first suspected.* The mortality in this ill-fated ship was dreadful—upwards of seventy men died out of a frigate's crew! No contagious character was evinced in this case.—Not so in the next.

4. The Scout sloop of war, with a crew of 102 men, arrived in Port-Royal on the 13th May 1822, from England, in health. Here, however, there was some intemperance, and a good deal of exposure to the sun by day, and dews by night.

In the course of three weeks nine were sent to the hospital with fever, of whom four died. The Scout sailed on the 14th June and arrived at the Havannah on the 28th. During this passage from five to seven cases of fever were daily added to the list. At one time there were upwards of thirty confined to bed, most of them with the black vomit and delirium. We have seen the surgeon's journal—and well might he say—"Human eye cannot have witnessed greater wretchedness and misery than existed on board at this time." We are very sorry that Dr. Burnett did not state the actual proofs of contagion evinced by the fever in this ship, because they are equally authentic, and if possible, more unequivocal than those exhibited in the Bann and at Ascension. We have seen the original documents and can vouch for the highly contagious character of the fever. Dr. Burnett is also *perfectly* satisfied on this point. "The instances of contagion," says he, "mentioned by the surgeon, appear to me so unequivocal that it is altogether unnecessary to say a word on this head." We do not think so—for the anti-contagionists are just as obstinate, and require just as strong facts to open their eyes as the ultra-contagionists. In this small vessel thirty-one died out of a crew of 102!

Dr. Burnett concludes with some observations on the two prominent symptoms, yellow suffusion and black vomit, which are well deserving the attention of the controversialists on both sides. If the *first* be taken as a criterion of yellow fever, Dr. Burnett can aver that, in the Mediterranean, he has seen many

---

* "We have the most decisive proof of the innocuous nature of coal-tar in the case of the Owen Glendower frigate, commanded by the Hon. Captain Spencer; she, being injected in the same manner as the Pyramus, proceeded to the South Seas, and after an absence of more than three years returned to England, having only lost one man, from a chronic complaint, who was in ill health before the ship left this country.

"At the different dock-yards where the process has been carried on to a considerable extent, no effect prejudicial to the health of the people has been produced, and the same may be said with respect to those employed in procuring this article from coals. Dr. Paris has been so kind as to examine some air taken from the pump-well of the Pyramus in the West Indies several months ago, and found it as pure as common air."

hundred instances where the yellow suffusion became "darker and darker," without the disease exhibiting any symptoms of contagion whatever.

"But we have different versions respecting the yellow suffusion, and in an account of this disease, as it has prevailed at Gibraltar, which a late writer praises as the best he has seen, we are told that 'when the disease terminates favourably, it is rarely attended with yellowness of skin, which if it does take place is of a very pale lemon colour.'

"This was certainly not the case with the two last epidemics which afflicted that garrison, and, I have reason to think, will not be found correct as applying to the fevers of the West Indies in general. My own opinion is, that yellow suffusion forms no distinctive mark of yellow fever. It is a common attendant on the febrile diseases of all warm climates or seasons. It has been observed long ago, in summer, amongst the marines exercised on South Sea Common, in the neighbourhood of the Morass, near Portsmouth, as mentioned by the late Doctor Lind; and more recently amongst the marine artillery, who suffered so much from remittent and intermittent at Fort Monckton.* I have seen it also in cases of purely sympathetic fever. The truth is, that the yellow suffusion is more or less dark in some, and altogether wanting in others, and hence, preserving no regular appearance, cannot be taken as a pathognomonic symptom, and though the word 'yellow fever' may produce an appalling influence on the mind, from the associations connected with that terrible name, yet, when viewed in all its bearings, we find that in more temperate climates it may form a leading symptom in fevers of a comparatively mild description.

"As to the black vomit, every one knows it is almost an inevitably fatal symptom. The recoveries after this has taken place, are indeed 'few and far apart,' and I readily acknowledge, that it is a much more frequent attendant on the fevers of the West Indies than on those of the Mediterranean; for, in the former, the disease generally runs its course more rapidly, often leaving but little opportunity of employing curative means, and resisting every mode of treatment; while, in the latter, more time is generally given, and hence the disease is not nearly so fatal; yet even here I have seen deaths as early as the third day, with all the aggravated symptoms attending the fatal cases of tropical fevers, viz. yellow suffusion, black vomit, and speedy putrefaction of the corpse."—p. 47.

Here we must take leave of Dr. Burnett, referring to his very candid and sensible report for various interesting particulars

---

* "The yellow fever, according to Lafuente, a celebrated Spanish physician, broke out in Medina Sidonia in 1801, when all the sea-port towns of Andalusia enjoyed the most perfect health. This city is situated at least thirty miles in the interior of Andalusia, and is a proof of the local origin of the Spanish epidemics, which no sophistry can overturn."—See Burnett on the Mediterranean Fever, 2d Edition, page 491.

which we cannot state in this place. No practitioner proceeding to a tropical climate should be without this official report, which contains most important facts and data that cannot be too carefully treasured up in the mind.

We now come to Dr. Mills's publication. We very much respect this gentleman for his zeal and talents; but we cannot blind ourselves to the fact that the worthy Doctor has got a crotchet in his head which sets all his music out of time. Upon this false note Dr. Mills has thrumbed from beginning to end of his book. Every body knows the noise that has been made in the world for nearly twenty years past, respecting the identity of fever and inflammation;—one party placing the seat of the phlogosis in the head, another in the stomach or bowels, and a third in some other fancy organ. Dr. Mills has chimed into the same path, and seemingly unmindful of Ploucquet, Marcus, Clutterbuck, Broussais, and a host of others who have preceded him, hopes "that the *new views* here taken of fever, and the *various lights* in which it is placed, may serve to render our ideas in respect to its nature and mode of treatment *clear and satisfactory*."* Now, we do aver that, were Dr. Mills's new views admitted and acted on, chaos would come again. But there is not the smallest danger of such an event. Our author, after much study, no doubt, discovered that all severe inflammations, especially of internal organs, were accompanied by *fever*. It was only necessary, therefore, by a little gratuitous assumption and strained analogy, to prove that all fevers *were* inflammations, differing not in their nature or treatment, but only in their seat.

To begin at the beginning then, the first parallel is naturally between phrenitis and typhus. In *both* there is head-ache, delirium, heat of skin, frequency of pulse, &c. *Ergo*, they are both the same disease. Just as Dr. Mills had finished his triumphant parallel, the unlucky idea of *contagion*, as an attribute of *typhus*, crossed his mind, and also its occasional *epidemic* character. Such stumbling blocks are nothing in Dr. Mills's way. "On these points, however, a diversity of opinion exists, and some perhaps will go so far as to say, that these distinctions are more imaginary than real." Thus the stubborn *facts* which we have brought forward in the early part of this

* We cannot conceive how Dr. Mills could be so indiscreet as to claim the title of "new views" for a doctrine of fever, precisely similar to that of Ploucquet, who, more than twenty years ago, averred that fever was sympathetic of inflammation of the brain, stomach, intestines, &c. and hence divided typhus into cephalic, gastric, splanchnic, &c. &c.

paper respecting contagion, are swept away by a single touch of the brush of sophistry.

The parallel between erysipelas and typhus affords our author great satisfaction. Erysipelas appears to be typhus in the skin—and typhus is erysipelas of the brain or its membranes.

There are not many who would institute a parallel between ophthalmia and typhus. In ophthalmia there is head-ache—so there is in typhus. There is intolerantia lucis in both—in short ophthalmia, according to the "new views," is typhus of the eye.

"Again, the symptoms proper to Ophthalmia, as, intolerance of light, pains in the eye-balls, suffusion of the eyes, and even loss of vision, occur not infrequently in Typhus: and it is well worthy of our notice, that, whereas, in Ophthalmia the inflammation, spreading from the eye to the brain, produces the symptoms of Typhus; in Typhus, the reverse of this takes place; the affection of the eye proceeds from that of the brain, showing the extension of the inflammation from an internal to an external organ." p. 11.

Finally:—

"Such is the comparison I would institute between Ophthalmia and Typhus, diseases which, on a superficial view, appear not merely different, but even opposite, in their characters; yet, when more closely examined, are found to be essentially the same." p. 12.

By similar chains of reasoning, Dr. Mills makes out to his own entire satisfaction the identity or great similarity of pneumonia and typhus—of enteritis* and typhus—of puerperal fever and typhus—of hepatitis and typhus—of gastritis and typhus—of small-pox and typhus. Our readers will not expect us to waste time in commenting on these forced comparisons and over-strained analogies. We are extremely sorry to see such a man as Dr. Mills employed on such hopeless undertakings.

It is well known that, to the advocates for the identity of fever and inflammation, ague or intermittent formed the most troublesome stumbling-block. The numerous disciples of Broussais have been for years endeavouring to explain away this phenomenon—and have, at length, effected their purpose, by discovering a class of *intermitting inflammations* to correspond with *intermitting fevers!* Dr. Mills appears either not to be acquainted with this discovery of the continental anti-entologists,† or else it is not quite satisfactory to him—he there-

---

* Dr. Mills is always quoting Dr. Mosely. Has he forgotten that that sapient physician adopted the idea of Sydenham, that "dysentery was a *fever* turned in on the *guts*." So much for these "new views."

† Those who maintain that fever may exist independent of a local inflammation, are branded by the disciples of the new light with the name of *Entologists*.

fore takes, in this instance at least, a "new view" of the affair, and boldly denies the existence of such a thing as an intermittent fever! "If," says he, "fever ceases, and then returns after an interval of one, two, or three days, *whither does it go, and whence return?* Can fever be absent and present at one and the same time?" These are curious questions. Does Dr. Mills suppose that fever is an entity which exists separately from the body? Is it not an assemblage of phenomena—which assemblage may cease for a time and then be renewed, without any great violence to reason or credibility. Will not a headache, a colic, and many other complaints cease for a time, and then be renewed? But leaving reasoning aside, we join issue with Dr. Mills on a matter of fact which every medical practitioner can decide upon by the evidence of his own senses. Dr. Mills asserts that there is no *intermission* of fever in an ague, but only a *remission*—in short, that the word apyrexia in this disease is a solecism. Now we appeal to those who have seen ague on a large scale, and especially after it has continued for some time, whether they have not observed numerous examples of complete apyrexia between the fits—where the person ate and drank heartily on his good days—had a quiet pulse, cool skin, and every secretion and excretion regular? The fact is incontrovertible. What respect therefore can we have for a man's theories who, in our faces, denies facts of the most clear and evident kind—and that for the purpose of bolstering up a clumsy hypothesis which cannot stand without crutches.—Really we have no patience with such trifling. Of what use is it for Dr. Mills to occupy many pages in proving, what has been known and observed since the days of Hipprocrates, namely, that an intermittent will change into a continued fever, and continued fever into an intermittent. We do not affirm that intermittent and continued fevers are essentially different in their natures. All we contend for is, that they do not necessarily depend on (though they may occasionally be accompanied by) a local inflammation. All Dr. Mills' cases and commentaries on the identity of the two kinds of fever, are completely thrown away. They prove nothing towards his hypothesis.

At page 71 we find our author congratulating himself on the change which has taken place in the treatment of fever, modestly presuming on his own instrumentality in the happy revolution. We shall only hint our belief (a belief grounded on some acquaintance with what is going on in the medical world) that the doctrines maintained by those who identify fever and inflammation, have led to a system of ultra-depletion which is now producing a recoil in the public mind that bids fair to end in a complete counter-current, and consequent return to the old system of debility and putrescency. This has ever been, and ever will be the case, when men run into outrageous extremes. Such extremes must produce their own remedies—and, as in many other instances, the remedy may be nearly as bad as the disease. We rely on the good sense, however, of the profession, and hope that the great majority will avoid the dangerous extremes of viewing fever as always depending on inflammation on the one hand, or debility on the other. The practitioner should come to the bedside of a patient in fever without prejudice. He should examine the condition of the organs and functions—relieving those that labour most by local or general depletion; but always bearing in mind that there are many fevers which will run a course in spite of all medicine, and consequently, that we are not to squander away the blood of the patient at the beginning, with the fallacious view of cutting short the disease. Nothing can authorize *profuse* bleeding in fever unless there be *unequivocal inflammation* of an organ, revealed by the common characters that proclaim inflammation under other circumstances. We are well aware, indeed, that in all, or almost all fevers, there is some one organ or part that suffers more or less of inflammation or congestion, from previous susceptibility. But we also believe that, in a great majority of these cases, a constant attention to the functions of the bowels and skin will conduct these slight phlogoses to a safe termination. In this disease, dissection, like most other good things, has caused some evil, and led into some error. The consequences of fever have been set down as its causes. This is a point of danger which we have often descanted on—and we find one of the ablest pathologists of the age in which we live, inculcating the same doctrine. The organic changes which we find after death from fever, Laennec affirms are "*evidemment posterieures a la fièvre*, et ne peuvent, par consequent, être regardées comme sa *cause*, quoiqu'elles puissent quelquefois en augmenter l'intensité ou produire une veritable fièvre *secondaire et symptomatique*."* It is our firm belief, that it is this *secondary or symptomatic fever* which has led Dr. Mills and others into all the mazes of error through which they have run, and through which they have dragged so many others. The doctrine is replete with danger in the hands of inexperience, and calculated to bring a rational system of treatment into unmerited disrepute. We repeat our belief that

* See Laennec's Hospital Report in the REVUE MEDICALE for May 1824, page 163.

idiopathic fever has an existence—that it is not *caused* by local inflammation—and that the *latter*, when it does take place, is only an accompaniment or contingency, and not at all the essence or source of the fever.

Dr. Mills has given us several short distinct essays on fever, its causes, and its treatment, which contain much judicious advice, but still tinctured with the leaven of erroneous doctrine that pervades the whole work.

At pages 111–12–13, Dr. Mills has embodied fifteen *reasons* "for considering fever not infectious." We shall oppose to these fifteen *reasons*, one solitary *fact*—the catastrophe at Ascension. Let the public judge between us.

And this naturally brings us to Dr. Maclean's work. We are disposed to think much better of this extraordinary writer than we did a few years ago. For a long time, we suspected that Dr. Maclean was writing for notoriety, and preaching doctrines and opinions (like some of the priests whom he ridicules) in which he puts no credence himself. We are now convinced that there is a dark speck in his mind's eye, in consequence of which he could not see *contagion*, even if it presented itself in the tangible shape of a brace of buboes in his own groins. A man in such a state of adumbration is rather to be pitied than censured:—and, as his lucubrations have long ceased to make the slightest impression on the medical profession, it is but charitable to let the author have the full enjoyment of thinking and proclaiming himself more enlightened than all his brethren put together. We have no doubt that monomaniacs feel a greater pride and pleasure in maintaining their delusions, than philosophers ever experienced in evolving the most sublime truths, or men of science, the most important discoveries—in other words:—

> Whatever Nature has in *sense* denied,

> She gives in large supplies of useful *pride*.

Thus we see that human happiness is nearly balanced, even between those wide extremes, the fool and the philosopher—a fact that may well humble the vanity of man, while it proclaims the wisdom of his Creator!

But to return to Dr. Maclean. We have absolved this author from all *intention* to deceive or mislead the public—he himself being the misled and deceived. We also give him credit for unconquerable zeal—a zeal which we have reason to believe, ran counter to his best interests, and was subversive of his prospects in this country. So far he appears to have acted on principle—and that a disinterested principle. But, alas! zeal, principle, good intentions, are not always connected with good or useful pursuits—they are too often enlisted under the banners of error! The venerable Jackson and the eccentric Maclean left their homes to wander through countries scourged with plague and pestilence, where their lives were daily hazarded in the cause of science. Their object was *apparently* the same; but the modes of attaining it were very different. Dr. Jackson travelled in search of truth—Dr. Maclean, to confirm a preconceived opinion, and that an erroneous one. The former saw both sides of the question, and has recorded what he saw with simplicity and candour. The *latter*, either shut his eyes upon, or, as we hinted before, was completely blinded to, every fact that militated against his favourite doctrine. The consequence has been, that, from Alpha to Omega of Dr. Maclean's researches and investigations, we see nothing but the special pleader carefully setting before us one side of the picture dressed out in all the colours, tropes, and figures of oratory; while the other side is most studiously kept concealed from our view, or, if unavoidably exposed, so distorted and bedaubed as to be scarcely recognizable. We shall quote a passage from Dr. Maclean himself, which we have no doubt will justify the foregoing remarks. It is well known that our author, full of chivalrous valour in his crusade against the contagion of plague, entered the Pest Hospital at Pera, convinced that the enemy he pursued was a mere phantom of the imagination. *Five days afterwards*, a brace of buboes in his groins announced to him that he had caught a tartar. In fact, he was seized with the plague. Now whether the presence of this disease opened his mental eye for a moment to the light of truth—or that he was seized with a transient fit of candour—or, which is most probable, that his vanity would not permit him to conceal so formidable an adventure as that of grappling with the plague in a Greek pest-house—but so it was, that he *did* record a fact which damned his own theory. Dr. Paris, in his Medical Jurisprudence, took occasion to advert to this fact, in the following rather sarcastic language. "What can be the *organization* of that man's *mind*,* who goes into the Greek pest-house at Constantinople, and, according to his own statement, is attacked *on the fifth day* after he entered it, with the plague, and yet continues to assert that the malady is non-contagious?" Now let us see how our special pleader makes out this accident in the pest-

* By the way we are rather surprised that so logical and orthodox a writer as Dr. Paris should talk of the *organization of the mind*. If the mind be *organized*, it must be composed of *matter*—and such doctrine must be the doctrine of materialism.—*Rev.*

house to be a *proof of the non-contagious character of plague*!! It is a comment on Dr. Paris's paragraph.

"Differences of conclusions, I suspect, must depend rather upon mental furniture, than organization; and the phænomenon which appears to have excited so much suspicion in the minds of so many of my learned colleagues, might probably admit of a solution by a slight craniological process. If on the one side we should find the protuberances of pestilential contagion, whether of cholera, plague, yellow fever, typhus, dysentery, diarrhœa, or even scurvy, in full vigour, while on the other side they had either never existed or are wholly obliterated, will not such a difference naturally account for the greatest possible difference in conclusions? But without waiting for the *post mortem* appearances, which can only be of use to our successors, let us examine this phænomenon by the lights of our reason, upon the grounds that are actually before us. The proposition of my commentators, as far as it can be analysed, is, that a plague which attacks a person in a *Greek* Pest Hospital *at Constantinople, on the fifth day* of his residence, *must* depend upon *contagion*, and *cannot* depend upon *any other cause*. Now, *causâ argumentandi*, I will grant them all this; and then let us see what will be the consequences. There were twenty persons in health, or in a few instances with slight ailments, who had resided for months or years in the hospital, and were in daily communication with a succession of pestiferous patients, yet had not the disease: but if twenty persons in health, or with slight ailments, can be for months or years in daily intercourse with pestiferous patients, without becoming pestiferous, whilst one only who was so circumstanced, was affected; is not the conclusion inevitable, that the disease is not contagious? Here then, supposing that the fact of contact were evidence of contagion, there would be *twenty to one* in respect to number, without reckoning time, against the disease by which I was affected at the Greek Hospital, being the product of a specific *virus*." 380.

Setting aside the ill-timed attempts at wit and humour in the above passage, where there should have been nothing but serious argument, what will our readers think of Dr. Maclean's inference, that, because twenty persons who had been for months or years *accustomed* to the contagion of plague escaped, while he (a fresh man) was quickly affected, it is twenty to one against the plague being contagious! Yet these are the kinds of argument brought forward through a great part of his work—arguments that might do very well for a wrong-headed reformer in the House of Commons, but which must greatly injure a medical writer in the eyes of his brethren—the only legitimate judges.

In respect to the quarantine laws, every well-informed man, we believe, wishes their revision, and deprecates many of the restrictions which are laid on commerce, not only here, but in the Mediterranean, where they are ten times more vexatious. We conceive that Dr. Maclean has rather retarded than advanced this desirable change, by the intemperate manner in which he attacked the settled opinions of his brethren as well as the laws of his country. This was not the way to procure legislative ameliorations, as the event has shewn. It is now quite useless to attempt any appeal to parliament for years to come, after the disclosure of the visionary and erroneous doctrines which Dr. Maclean forced on the notice of the national council, the College of Physicians, and the profession at large. We hope, however, that the result of this enterprize will prove a warning to future adventurers.

It will be seen that Dr. Maclean has strangely, but somewhat artfully, jumbled together plague, yellow fever, typhus, dysentery, diarrhœa, cholera morbus, and even scurvy, ridiculing the idea of their being contagious. But no one ever dreams of coupling contagion with one half of the above list—at least none but a few septuagenarian drivellers in their second childhood, who ought to be playing with their rattles instead of entering the field of contention with their great grand children.

Finally, we have no hope that any thing we have said, or indeed that any one can say, on the subject of contagion shall awaken Dr. Maclean from the delusive dream, or "fool's paradise," in which he has lived so long. Notwithstanding that volume after volume rolls from his prolific pen, and lies, in its turn, neglected by the public, still Dr. Maclean is as sanguine as ever! In fact, as the following quotation will shew, he already considers himself as the victor, and that plague, pestilence, contagion, and quarantine, are prostrate beneath his feet.

"Had I perished in the course of my experiments in the Levant, it might with some plausibility have been said of me by these commentators, as Mr. Tully has said of Dr. M'Adam, that I fell a victim to my want of belief in the contagion of the plague; they would have been credited by the multitude, great and small; and epidemic diseases might have remained contagious, and sanitary laws have been employed, 'to *chain* those *hurricanes* of the human frame' for ages to come. But

"I survive,

To mock the expectations of the world;

To frustrate prophecies, and to raze out

Rotten opinion." 382.

Two small works still remain to be noticed in this article. The first is Dr. Stoker's Hospital Report. It presents a considerable contrast with the speculative production of Dr. Mills. It is principally occupied with numerical and statistical statements which cannot be analysed. We find from tables carefully composed, that the average rate of mortality in fever, from 1804 till 1824 in the Dublin Fever Hospital, was (in round numbers) as one in fifteen. The greatest mortality (in the years 1807–8–10 and 23) was one in eleven. The least mortality was one in thirty, in the year 1818—the year in which there was the greatest number of admissions. In the severe forms of genuine typhus fever—the average mortality has generally been one death to four recoveries—or one in every five admitted. Our author, who has had almost unparalleled experience in fever for more than a quarter of a century past, entertains sentiments somewhat different from his countryman, Dr. Mills, as will be evinced in the following extract.

"Simple Fever arises from morbid actions, which do not produce any change in the structure of parts,—*Symptomatic Fever* from morbid actions produced by change in the structure of parts,—and Typhus Fever from certain morbid changes, primarily in the fluids, which produce morbid actions, and sometimes change of structure, in the parts.—The relations which these three classes of fevers bear to each other may be illustrated by familiar examples in surgery,—the heat and redness produced by friction being taken to represent Fever; an incised or lacerated wound to represent Symptomatic Fever; and a wound by which poison or virus was introduced to represent Typhus Fever." 22.

Again :—

"The result of dissections, instituted with a view to the nature of Fever, by Dr. Beddoes, and by Mr. Kirby, and Dr. M'Cartney, in answer to my own and subsequently Dr. Barker's queries, corresponds with that of the *post mortem* examinations that I have witnessed; and, inasmuch as altered structure of parts was thus proved not to have taken place in numerous instances, on minute inquiry, it is quite conclusive, that altered structure cannot be deemed an essential characteristic of fever, a conclusion which cannot be affected by any subsequent evidence, however that may go to prove that Typhus Fever frequently supervenes on inflammatory diseases, or that it as frequently discloses organic affections which had been previously latent." 24.

Our author truly observes, that the advocates for the inflammatory nature of typhus fever have pursued their anatomical researches through every cavity of the body—from the brain and spinal marrow to the minutest distribution of the nerves. Each, in turn, has discovered in some instances what he looked for—"but as frequently each has overturned the opinion of the preceding inquirer respecting the seat of the fever." The following observations are rational enough.

"The great source of the discrepancy of opinions which has taken place amongst those engaged in *post mortem* examinations, respecting the nature and seat of Typhus, has been, as I suppose, that at certain seasons of the year, and peculiar states of the weather, in which inflammatory diseases prevail, and inflammation, as is well known, falling in certain seasons and conditions of the atmosphere, on some parts of the body more than others, then the local affections thus excited impress their characteristic marks on structure; these, possibly, are rendered more obvious when Typhus Fever supervenes, to the attacks of which the debility consequent on such inflammatory distempers, or the incident confinement in the miasmatous damps of the dwellings of our poor, render them peculiarly susceptible." 25.

For the numerous cases detailed by Dr. Stoker, his remarks on the humoral pathology, his tables, and statistics, we must refer to the little volume itself, which contains a great deal of practical information.

Mr. Wade's brochure is "expressly intended for the notice of students," and they will certainly do well to peruse it. The author properly observes that "blood-letting has great charms with most students:—there is at once a boldness and decision in the remedy which pleases them—besides it is surgical."

"It has appeared to me of late, that their great error has been to treat fever too much (more) like a mere local inflammation than as a disease which is probably to last for two or three weeks, and perhaps longer." vi.

Truly this is not the error of students alone. They would not commit it did they not see their masters do the same. The little work in question is calculated to disenamour the student of particular theories imbibed from particular teachers and writers. It inculcates rational conduct—and evidently proves that the writer is possessed of good sense, and has exercised a considerable share of observation in the institution to which he is attached.

